# Bariatric Surgery in Cebu, Philippines: Current Status and Initial Experience With Laparoscopic Sleeve Gastrectomy

**DOI:** 10.7759/cureus.18953

**Published:** 2021-10-21

**Authors:** Ralph Victor Yap, Patrick John Eleazar, Vincent Matthew Roble II, Don Edward Rosello

**Affiliations:** 1 Department of Surgery, Cebu Doctors' University Hospital, Cebu, PHL; 2 Department of Surgery, Section of Minimally Invasive Surgery, Cebu Doctors' University Hospital, Cebu, PHL; 3 Department of Surgery, Section of Minimally Invasive Surgery, Cebu Doctors’ University Hospital, Cebu, PHL

**Keywords:** obesity, developing country, weight loss, philippines, laparoscopic sleeve gastrectomy, bariatric surgery

## Abstract

Background

The prevalence of obesity in the Philippines has increased more than three-fold over the last two decades. However, bariatric surgery has not been widely adopted yet in the country. Local data mainly on laparoscopic adjustable gastric banding (LAGB) and Roux-en-Y gastric bypass (RYGB) are limited as well. We report for the first time our experience with laparoscopic sleeve gastrectomy (LSG) and present the current local status of bariatric surgery in Cebu, Philippines.

Patients and methods

This is a retrospective study of all patients 18 years old and above who underwent LSG in a single, private, tertiary institution during the period 2009 - 2019. Our primary endpoint was weight loss after LSG. Secondary endpoint was postoperative complications.

Results

Thirty-three patients (mean age 40.9 ± 14.5 years) underwent LSG. Baseline weight and BMI were 112.6 ± 29 kg and 41.3 ± 8.6, respectively. The mean operative duration was 201 ± 72.9 minutes. The were no open conversions with minimal morbidity. Mean hospital stay was 3.7 ± 0.9 days. The postoperative mean weight and BMI after one year were 68.9 ± 17 kg and 26.6 ± 6, respectively. Overall, mean excess weight loss (EWL) was 61.9 ± 44.1 % at a median follow-up of 5.4 months. Significant weight loss was noted after the third month.

Conclusion

LSG is a safe and effective method in producing weight loss. It can be a definitive treatment option as local prevalence of obesity is increasing in the Philippines. However, access to and the practice of bariatric surgery remains limited in the country. A collaboration among private and government stakeholders is essential.

## Introduction

The prevalence of obesity has increased worldwide over the past 50 years reaching pandemic proportions as described by the World Health Organization. It ranges from 3.7% (Japan) to as high as 38.2% in the United States, varying from country to country [1]. In 2020, the prevalence of obesity among adults in the Philippines was 8.2%, a rate three times higher than in 1993 [2]. Despite still belonging to countries with a low obese population, the obesity prevalence in the Philippines has increased at an alarming rate [3].

The Philippines is an archipelagic country divided into three major geographical areas, namely Luzon, Visayas, and Mindanao. Its capital (Manila) is located on the largest island of Luzon where the majority of the level III (where complex/subspecialty surgeries are being performed) hospitals are located. Cebu province, on the other hand, is located in the central Visayas. It is 570 kilometers away from the country's capital and has the fifth most populated city (Cebu city) in the Philippines. Previous data has shown that the prevalence rate of obesity among adults in Cebu has reached 8.7%, which is slightly higher than the national rate [4]. Currently, Cebu is among the top three provinces with the highest obese population in the country.

Despite public health efforts aimed at obesity prevention and weight management, local data from the Cebu Longitudinal Health and Nutrition (CLHN) Survey from 1998 to 2015 have shown that the rates of overweight and obesity have markedly increased over the survey years [5]. Thus, substantial health and economic burdens are to be expected. Specifically, this will escalate the incidence of obesity-related coronary heart disease, dyslipidemia, hypertension, sleep apnea/respiratory problems, cancer, type 2 diabetes mellitus (T2DM), and health-care/medication costs.

While intensive lifestyle intervention and pharmacotherapy have been recommended as first-line treatment by major obesity treatment guidelines, their long-term effect on weight loss, T2DM remission, and reducing cardiovascular morbidity/mortality have been unfavorable compared to bariatric surgery. Common surgical procedures for obesity include laparoscopic adjustable gastric banding (LAGB), sleeve gastrectomy (LSG), Roux-en-Y gastric bypass (RYGB), and biliopancreatic division with duodenal switch. These procedures are considered more efficient in ameliorating obesity and its associated comorbidities [6]. 

Patients having a body mass index (BMI) of > 35 (class II obesity and above), and 30 to 34.9 (class I) with T2DM or other obesity-related diseases are candidates for bariatric surgery in many South/East Asian countries. In addition, the BMI threshold for Asian populations is reduced by 2.5 when metabolic surgery (for T2DM) is considered. In the Asia-Pacific Metabolic and Bariatric Surgery Society (APMBSS) 2018 survey, there were 30 bariatric surgeons registered out of 20 institutes in the Philippines. This translates to a ratio of one bariatric surgeon for every 300,000 obese adult Filipino population [2,3]. Secondly, there has been only one active surgeon performing bariatric procedures in Cebu since 2007.

In 2017, the Philippines logged the lowest number (55) of bariatric/metabolic surgeries in Southeast Asia. LSG and bypass surgery comprised 63.6% and 16.4% of the total procedures, respectively. While LSG has become the most commonly utilized bariatric procedure in the country, local data on its outcomes are lacking. To the best of our knowledge, there have been only two publications reporting on obesity surgery in the Philippines since 2013 [7,8]. They analyzed the results of LAGB and compared it with RYGB among different age groups in a private tertiary hospital. Thus, this study aims to (1) share for the first time our experience with LSG and its effect on weight loss among adult obese patients in Cebu, Philippines, and (2) present the current status of bariatric surgery at a tertiary hospital remote from the country's capital.

## Materials and methods

Study design

This is a retrospective study on all consecutive LSGs performed on obese patients aged 18 years old above from July 1, 2009, to December 31, 2019, at a 300-bed private tertiary hospital in Cebu City, Philippines. This study was conducted in accordance with the Declaration of Helsinki and the institutions’ research ethics committee approved the study (Protocol Code 2-2020-006). Written informed consent was waived due to the nature of the study design. The prospectively maintained surgical record database of the institution’s Department of Surgery was queried for the term “LSG”. The list of eligible patients was generated and their corresponding electronic medical records were reviewed.

Preoperative preparation

Preoperatively, medical clearance was sought from appropriate internists when clinically indicated. Cholecystectomy was routinely offered and performed concurrently with LSG based on patients’ preferences. Venous thromboembolism prophylaxis was given when indicated. The patients were positioned supine on the operating room table with a footboard and straps. Compression stockings were applied and both upper extremities were tucked on the sides. An intravenous antibiotic was given before skin incision.

Surgical technique

All procedures were performed by a single surgeon (D.E.R.) experienced in both upper gastro-intestinal and bariatric surgeries under general endotracheal anesthesia. The surgeon was positioned on the patient’s right-hand side while the surgical assistant stood on the opposite side. Initial trocar access (12-mm optical port) was achieved using an open Hasson’s technique at the supra-umbilicus. Pneumoperitoneum was maintained at an insufflation pressure of 13 mmHg. Under direct vision, two 5-mm ports were placed on the right subcostal area, and below the xiphoid process as a left-hand port and for insertion of a Nathanson liver retractor, respectively. The fourth trocar (10-mm) was placed at the left subcostal area as a right-hand port. The operating table was then placed in a reverse Trendelenburg position.

While retracting the left lobe of the liver, initial dissection was initiated at the midbody of the stomach along the greater curvature. The gastroepiploic and short gastric vessels were divided using a Harmonic scalpel (Ethicon, Johnson and Johnson, Parañaque, PH) and the lesser sac was entered. The stomach was mobilized up to the angle of His, dividing the posterior pancreatic attachments and exposing the left crus of the diaphragm. Caudally, the dissection was carried until the proposed site of transection (4 - 6 cm proximal from the pylorus). A 38- to 42-French tapered bougie was then passed through the mouth into the stomach and positioned along the lesser curvature. The laparoscope was repositioned through the left subcostal port and the reticulating linear stapler was inserted at the supraumbilical port. The linear stapler was positioned parallel to the lesser curvature and close to the bougie while avoiding narrowing of the incisura.

Resection of the greater curvature (up to 70% to 80% of the stomach) began caudally using appropriate stapler heights up to 2 cm lateral to the angle of His and away from the gastroesophageal junction cranially. The bougie was removed and the staple line was plicated with a continuous Polydioxanone 3-0 suture. Cholecystectomy was then performed. The resected stomach and gallbladder specimens were extracted using a retrieval pouch through the supra-umbilical port under direct vision. Placement of a Jackson-Pratt (JP) drain along the stomach and passed through the left subcostal port is preferred in patients with a baseline BMI of > 40.

Postoperative care

The patients were placed on nil per os (NPO) postoperatively. A gastrogram was performed on the first operative day. If no leaks are detected, a general liquid with supplemental enteral nutrition was started and continued for three weeks. If a JP drain was placed during surgery, the leak test was performed by letting the patient ingest a sufficient amount of a diluted methylene-blue solution instead of a gastrogram. The JP output is monitored within eight hours for blue-stained fluid (leak positive). The drain was removed on the third postoperative day before home discharge. Patients were advised to follow up after three weeks as the diet was progressed, and every three months for the first year then every six to 12 months thereafter.

Outcomes

The variables investigated from the medical records of the patients included age, gender, American Society of Anesthesiology (ASA) score, comorbidities, prior abdominal surgery, baseline weight and BMI, the performance of cholecystectomy, operative duration, intraoperative complications, in-hospital morbidity and mortality, and length of hospital stay. Additional data on the patients’ follow-up outcomes (i.e., weight change, and procedure-related complications) were collected from the medical records at the outpatient clinic of the bariatric surgeon. Due to patients’ variability in compliance to follow-up schedules, three time periods (<three, three to 12, >12 months) were used to compare the postoperative weight and BMI from baseline and the percentages of total (%TWL) and excess (%EWL) body weight losses. A BMI of 24.9 was used as standard reference for an “ideal” body weight. The lowest weight and BMI attained within the three follow-up intervals were used. All collected data was encoded in Microsoft Excel (Microsoft Corp., Redmond, WA, USA).

Statistical analysis

All values were expressed as mean ± standard deviation and/or median (range) for continuous variables, and frequencies (percentages) for categorical variables. Mean values were compared using Student’s t-test and/or one-way analysis of variance (ANOVA) where appropriate. A P value of less than 0.05 was considered statistically significant. All statistical analysis was performed using the IBM SPSS Statistics for Windows, version 26.0 (IBM Corp., Armonk, NY, USA).

## Results

A total of 33 consecutive patients with a mean age of 40.9 ± 14.5 years underwent LSG during the study period, of which 67.7% were females. The majority (78.8%) of the patients had an ASA score of 2. The two most common comorbidities were hypertension and T2DM while 24.2% of patients had previous abdominal surgery (two with prior LAGB who underwent concurrent gastric band removal and LSG). The baseline weight and BMI of all patients were 112.6 ± 29 kg, and 41.3 ± 8.6, respectively. The mean operative duration for all cases was 201 ± 72.9 minutes. Cholecystectomy was performed concurrently with sleeve gastrectomy in 66.7% of the cases. However, no significant difference was found (p = 0.687) in terms of operative time between the LSG only and LSG with cholecystectomy groups (Table 1).

**Table 1:** Baseline characteristics of patients * = Mean ± standard deviation. † = p-value comparing LSG with LSG + LC. Abbreviations: ASA = American Society of Anesthesiologists physical status classification; HPN = hypertension; T2DM = type 2 diabetes mellitus; BMI = body mass index; LSG = laparoscopic sleeve gastrectomy; LC = laparoscopic cholecystectomy; OSA = obstructive sleep apnea

	n = 33	%
Age (years)*	40.9 ± 14.5	--
Sex		
Male	11	33.3
Female	22	67.7
ASA Score		
1	6	18.2
2	26	78.8
3	1	3
Co-morbidities		
HPN	13	39.4
T2DM	13	39.4
Dyslipidemia	9	27.3
OSA	3	9.1
Previous abdominal surgery	8	24.2
Baseline weight (kg)^*^	112.6 ± 29	--
Baseline BMI^*^	41.3 ± 8.6	--
Operative Duration (minutes)^*^	201 ± 72.9	--
LSG only (n = 11)	208.4 ± 52.6	0.687^†^
LSG + LC (n = 22)	197.3 ± 82.1	

There were no conversions to open surgery. Three patients developed nausea and vomiting, atelectasis, and heart failure in the postoperative setting, respectively. The mean hospital stay after surgery was 3.7 ± 0.9 days. There were no occurrences of staple line leak, bleeding, stenosis, and surgical site infection. Likewise, there were no reports of re-admission, re-operation, nor mortality within the 30 postoperative days (Table 2). Out of 33 patients, only 23 (69.7%) had follow-up weight loss data.

**Table 2:** Postoperative outcomes/complications * = Mean ± standard deviation. Abbreviations: LOHS = length of hospital stay

	n = 33	%
Conversion to open	0	0
Complications		
Staple line leak	0	0
Bleeding	0	0
Nausea and vomiting	1	3
Stenosis	0	0
Surgical site infection	0	0
Atelectasis	1	3
Heart failure	1	3
LOHS^*^	3.7 ± 0.9	--
Re-admission	0	--
Re-operation	0	--
Mortality	0	--

The follow-up compliance rates were 66.7%, 39.4%, and 21.2% within < three, three to 12, and beyond 12 months from surgery. The mean duration among the three follow-up intervals were 1.7 ± 0.7, 6.8 ± 2.5, and 30 ± 15.1 months, respectively. The mean postoperative weight was 104.2 ± 27.7 kg, 81.5 ± 36 kg, and 68.9 ± 17 kg, respectively at the different follow-up periods. While the mean postoperative BMI were 38.5 ± 8.5, 31.7 ± 9.5, and 26.6 ± 6, respectively. Compared to baseline, significant differences in the postoperative weight and BMI were noted after the third month of follow-up (p = 0.011, 0.002). The mean %TWL and %EWL were 20.8 ± 11.6, and 61.9 ± 44.1, respectively at a median follow-up of 5.4 months (range 0.2 to 46.4). An %EWL of > 100 (mean 126.5 ± 23.9%) and a BMI of < 24.9 (mean 22.7 ± 1.9) were achieved by six (18.1%) patients. There was a significant increasing trend in the %TWL and %EWL at the different follow-up intervals (ANOVA, p = 0.000) (Table 3).

**Table 3:** Weight loss after laparoscopic sleeve gastrectomy * = Compared to baseline. † = at a median follow-up of 5.4 months (range 0.2 – 46.4; IQR 2, 14.9). Abbreviations: kg = kilograms; SD = standard deviation; BMI = body mass index; TWL = total weight loss' EWL = excess weight loss

	Mean ± (SD)	p-value
Post-op weight (kg) at different follow-up intervals*		
< 3 months	104.2 ± 27.7	0.286
3 – 12 months	81.5 ± 36	0.011
> 12 months	68.9 ± 17	0.003
Post-op BMI at different follow-up intervals*		
< 3 months	38 ± 8.5	0.183
3 – 12 months	31.7 ± 9.5	0.002
> 12 months	26.6 ± 6	0.000
%TWL at different follow-up intervals		
< 3 months	11.2 ± 4.5	0.000
3 – 12 months	25.2 ± 9	
> 12 months	31 ± 7.6	
Overall %TWL^†^	20.8 ± 11.6	--
%EWL at different follow-up intervals		
< 3 months	31.7 ± 17.1	0.000
3 – 12 months	77.8 ± 43.1	
> 12 months	104.1 ± 41.6	
Overall %EWL^†^	61.9 ± 44.1	--

## Discussion

In our study, two out of eight patients with previous abdominal surgery (PAS) were failed LAGBs which required concomitant band removal and conversion to an LSG. A one-stage conversion has shown to be safe and does not increase postoperative complication rates. Additionally, the presence of intra-abdominal adhesions appears to increase significantly in a two-stage conversion. However, careful preoperative assessment of patient eligibility for a one-setting conversion is necessary [9]. PAS has been traditionally considered a risk factor for higher open conversion and complication rates, and an increased operative duration when contemplating a laparoscopic procedure. A cohort study comparing 777 (with PAS) to 532 (without PAS) patients who underwent LSG did not show a significant difference in their median operative time. The overall postoperative complication rate was also similar. It concluded that LSG is safe to perform in obese patients with PAS and its presence does not constitute a contraindication to bariatric surgery [10].

The role of concomitant cholecystectomy (CC) during LSG in obese patients even with an established asymptomatic gallstone disease remains a controversial topic with no consensus among bariatric surgeons. More than 40% of obese patients without preoperative evidence of gallstone disease who underwent LSG alone may eventually develop gallstones within 18 months up to an average of 7.5 years. Among these cases, the rate of symptomatic cholelithiasis with subsequent cholecystectomy ranges from 31.8% to 36.9% [11,12]. A similar study in an Asian obese population also showed a rate of 27.5% new gallstone formation at one year. The rate of subsequent cholecystectomy after LSG varies in different studies but is generally considered low [13]. Moreover, CC during LSG can significantly prolong the operative duration by 27 to 33 minutes. In a retrospective analysis, CC may increase the risk of bleeding and postoperative pneumonia. However, this was not seen in propensity-matched cohort analysis of 4048 LSG patients except for a slightly increased risk of surgical site infection (SSI). In general, CC is not associated with an increased risk of major complications [14]. The performance of CC regardless of gallbladder status and symptomatology may be dependent on institution/surgeon, and/or patients’ preference after preoperative counseling about the risks of gallstone formation and/or its consequences in long term. Considering that the Philippines is a low/middle-income country, its performance may not only prevent patient anxiety for a second hospitalization and operation under general anesthesia but also additional financial costs.

The mean operative duration of all consecutive cases in our study (n = 33) of LSG was 201 ± 72.9 minutes. Cumulative-sum (CUSUM) analyses have shown that 58 to 68 LSG procedures may be necessary to achieve skill proficiency [15]. In a systematic review, a three-phase learning curve model was introduced where proficiency and mastery in LSG would require between 60 to 100 and 100 to 200 cases, respectively [16]. We expect our operative time to decrease as well in the future as our cases of LSGs further build up in the institution.

Common procedure-related complications of LSG include staple line leak and bleeding, stenosis, and gastroesophageal reflux disease (GERD). A randomized clinical trial comparing LSG and LRYGB has shown that both procedures had similar rates of early and late complications in a five-year follow-up. Reoperation rates were similar as well [17]. A 2015 MBSAQIP database analysis comparing 93,062 LSG and 41,080 LRYGB has shown that the former had a significantly lower incidence of a postoperative leak, serious morbidity, and mortality. However, the most common indication for reoperation in the LSG and LRYGB groups were severe GERD, and internal herniation, respectively [18]. Up to 30% of patients who underwent LSG may eventually require revisional surgery due to procedure-related complications, weight regain, inadequate weight loss, and development/worsening of GERD [19]. Options include re-sleeve gastrectomy, conversion to LRYGB, duodenal switch, or single anastomosis duodeno-ileal bypass. However, these more complex revisional procedures can be associated with increased postoperative complication rates. In our limited experience, there were only minor complications with no mortality.

A retrospective, multicenter study compared LAGB, LSG, and LRYGB (n = 735 patients) in terms of effectiveness and safety at a mean follow-up of 3.1 ± 1.2 years. LAGB achieved less %EWL, as well as higher complication rates compared to LSG and LRYGB. At one year follow-up, LSG achieved a significantly higher mean %EWL than LRYGB (76 ± 23 % vs. 71 ± 20%). However, no significant difference was observed thereafter [20]. In a 2017 meta-analysis, LSG was found to produce more %EWL at > five years when compared to LAGB (47.9% vs. 53.25%) but is lower than that of LRYGB (62.58%) [21]. A recent 11-year experience comparative study involving 396 LRYGB and 750 LSG procedures did not find any differences in weight loss and weight regain after the eighth year. Secondly, severe complications, including surgery-related mortality were not statistically significant between the two groups [22]. This shows that LSG seems to be an appropriate alternative as a definitive bariatric procedure. Successful obesity surgery has been traditionally defined as achieving and/or maintaining > 50% EWL. In our study, LSG achieved a mean %EWL of 61.9 ± 44.1, similar to previously mentioned international data, but at a limited follow-up period.

LSG has been shown to provide substantial and immediate glycemic control in patients with T2DM. A prospective study comprising 226 T2DM patients with a BMI of > 35 showed a rate of 38.1%, and 10.6% complete and partial remission of T2DM after LSG, respectively, at two years follow-up. On logistic regression, an age of < 45 years, and a short duration of T2DM from diagnosis were found to be independent predictors of complete remission [23]. A retrospective analysis of 143 obese patients diagnosed with hypertension who underwent LSG showed 23% and 63% complete and partial remissions of hypertension, respectively, at 12 months [24]. In a recent meta-analysis by Han et al. [25], both LSG and LRYGB are equivalent in overall T2DM resolution. Dyslipidemia and hypertension remission rates were also similar in both procedures at mid-term (12 to 36 months). However, LRYGB was more superior in controlling these comorbidities beyond the 36th month. The prevalence of young-onset diabetes (YOD) associated with class I/II obesity and/or metabolic syndrome has increased in the Philippines, and this makes LSG an attractive treatment option.

In this study, there was a decreasing trend of follow-up rates as the interval from surgery becomes longer. Poor follow-up compliance is a limiting factor in assessing short- and long-term outcomes after bariatric surgery not just in our locale/country but also in developed countries. Moreover, patients who complied with all scheduled clinic follow-up are more likely to achieve higher %EWL and %TWL in the long term [26]. Factors such as financial and patient-reported motivational issues are linked to non-compliance. Thus, the importance of follow-up should be strenuously discussed between health care providers and patients, and scheduled appointments preferably included in the insurance coverage [27]. Secondly, considerable and repeated efforts are needed to implement the proper documentation of surgery-related outcomes. The establishment of a local or national registry/database may be a good investment to create an estimate and demonstrate the safety of bariatric surgery practice in the country.

Current status of bariatric surgery in Cebu, Philippines

Currently, there are only two local obesity treatment guidelines readily accessible in the Philippines for more than a decade. A low-calorie diet, physical activity, behavioral, and pharmacologic therapies were the only therapeutic measures recommended for treating obesity by the UP-PGH Family Medicine Research Group in 2002. The Philippine Association for the Study of Overweight and Obesity (PASOO) algorithm for the healthy and safe weight management program in 2008 recommends referral to an inter-professional team when all these measures are not successful. This includes referral for “surgery” especially for patients with an extremely high BMI-related health risk [28]. However, our local surgical societies have yet to come up with guidelines for the utilization of bariatric surgery in our setting. There is a great need for updating and/or modifying the current sets of recommendations in the Philippines to arrive at an integrated approach to treat obesity based on available local data (Table 4) and resources [7,8].

**Table 4:** Bariatric surgery in the Philippines Values are expressed as mean ± standard deviation. * = Median (range). † = at mean follow-up of 30 + 15.1 months (21.2% follow-up compliance). LAGB = laparoscopic adjustable gastric banding; LSG = laparoscopic sleeve gastrectomy; RYGB = Roux-en-Y gastric bypass; SSI = surgical site infection; EWL = excess weight loss

Author (Year)	Number of patients	Procedure	Mean age (years)	Baseline Weight (kg)	Baseline BMI	Follow-up (months)	Follow-up compliance	Postop Weight (kg)	Postop BMI	%EWL	Remarks
Dineros [7] (2007)	50	LAGB (30)	38 ± 13.1	126.7 ± 25.4	48 ± 11.7	12	100%	65.9	22.2	31.7	Ventral hernia (20%); Band slippage (10%); SSI (4%); ICU admission (2%); Pneumonia (2%); Leakage (2%)
		Open (15) and Lap (5) RYGB						72.9	28.4	36.8	
Evora [8] (2013)	97	LAGB	36 ± 12.4	121.8 ± 30.9	44 ± 9.3	12 ± 6	68%	102.4 ± 25.8	36.7 ± 6.9	31.4 ± 19.3	Band explantation (5.1%)
Our study	33	LSG	40.9 ± 14.5	112.6 ± 29	41.3 ± 8.6	5.4 (0.2 – 46.4)^*^	69.7%	68.9 ± 17^†^	26.6 ± 6^†^	61.9 ± 44.1	Atelectasis (3%); Heart failure (3%); Nausea & vomiting (3%)

In our setting, the average cost (excluding preoperative evaluation and healthcare professional fees) for an LSG is 191,409 PHP (equivalent to 4,000 USD as of this writing). The Philippines’ National Health Insurance Program (PhilHealth) provides its members’ reimbursement for bariatric procedures (2014 updated RVS codes: 43842, -3, -6 to -8). Specifically, for LSG, members have entitled PHP 21,000 coverage for healthcare institution fees alone. This, however, only covers 11% of the average hospital cost in a private setting as previously mentioned. Secondly, private insurance providers in the country currently do not provide reimbursement for such procedures [3]. Access to and quality outcomes from colorectal cancer treatment and kidney transplantation have been improved lately in the Philippines with the successful implementation of the Z-package program by PhilHealth. Perhaps, the extension of such program to cover the necessary/major costs in the treatment of obesity will greatly benefit the obese population within the different regions in the country.

The initial bariatric procedures performed by D.E.R in our institution were endoscopic intragastric balloon placement and LAGB until 2009 where LSG was introduced and became the preferred procedure for obesity treatment. The average LSGs performed annually in our institution is only three cases. However, this does not include the cases being performed by D.E.R in two other private tertiary hospitals within the city. In contrast, the performance of bariatric surgeries in the United States has been centralized to high-volume centers to ensure “higher-quality” outcomes. To be accredited (level 2) as a Bariatric Surgery Center of Excellence, an institution must perform 25 or more bariatric cases annually and have at least one experienced and credentialed bariatric surgeon with a minimum of 50 bariatric cases performed in the previous two years. However, this concept of centralization may not readily apply to third-world and/or developing countries. Low-volume centers can demonstrate excellent outcomes by having a dedicated and well-trained bariatric surgical team, and a multidisciplinary team approach in perioperative management.

Other possible reasons why bariatric surgery has not been widely adopted yet in our region include lack of information dissemination to the general public, low level of awareness/knowledge about obesity and bariatric surgery among health care workers/primary care physicians (PCP), and the lack of bariatric surgery fellowship training within the country. Such factors have been included and discussed as current problems on obesity surgery in the recent APMBSS 2018 survey [3]. Despite numerous publications on the safety and effectiveness of bariatric surgery in producing durable weight loss and resolving obesity-related comorbidities, its penetration in the medical community remains limited. A recent survey among Swedish PCPs has shown that half of the respondents had high concerns about postoperative complications relating to bariatric surgery and this may lead to non-referral [29]. An individual’s choice to pursue bariatric surgery may also be influenced by familial involvement and support, and cultural and/or religious beliefs [30]. This study is limited by its retrospective design, poor patient follow-up compliance, and low sample size. This study also primarily relied on the only available bariatric surgeon within the city/province and to a single-center experience. Moreover, the authors were unable to collect data on the resolution of the obesity-related comorbidities from baseline among the included patients.

## Conclusions

Our study describes for the first time the outcomes of patients who underwent LSG for obesity, and the current scenario of bariatric surgery in Cebu, Philippines. Even with the limited number of cases, we have demonstrated that LSG provides effective weight loss (> 50% EWL) similar to international published data, and is associated with minimal morbidity. As the prevalence of obesity is increasing in the Philippines, LSG can be a definitive treatment option. However, the Philippines suffers the same hurdles that other developing countries are currently facing. Such that, access to obesity surgery in our region/country remains to be limited due to the multiple challenges surrounding the practice of bariatric surgery. Thus, a collaboration among private and government stakeholders/medical communities in the treatment of obesity is essential.
